# The pathophysiological significance of PPM1D and therapeutic targeting of PPM1D-mediated signaling by GSK2830371 in mantle cell lymphoma

**DOI:** 10.18632/oncotarget.11904

**Published:** 2016-09-08

**Authors:** Kensuke Kojima, Aya Maeda, Mariko Yoshimura, Yuki Nishida, Shinya Kimura

**Affiliations:** ^1^ Department of Hematology, Respiratory Medicine and Oncology, Division of Medicine, Saga University, Saga, Japan

**Keywords:** PPM1D, p53, MDM2, p38 MAPK, mantle cell lymphoma

## Abstract

PPM1D is a serine/threonine phosphatase that negatively regulates key DNA damage response proteins, such as p53, p38 MAPK, histone H2A.X, and ATM. We investigated the pathophysiological significance of PPM1D and its therapeutic targeting by the novel PPM1D inhibitor GSK2830371 in mantle cell lymphoma (MCL). Oncomine-based analyses indicated increased PPM1D mRNA levels in MCL cells compared with their normal counterpart cells. Higher PPM1D expression was associated with higher expression of the proliferation gene signature and poorer prognosis in patients. Eight MCL (three p53 wild-type and five mutant) cell lines were exposed to GSK2830371. GSK2830371 inhibited the cell growth, being prominent in p53 wild-type cells. GSK2830371 induced apoptosis in sensitive cells, as evidenced by induction of phosphatidylserine externalization and loss of mitochondrial membrane potential. p53 knockdown de-sensitized cell sensitivity. GSK2830371 increased the levels of total and Ser15-phosphorylated p53, and p53 targets p21 and PUMA. GSK2830371 and the MDM2 inhibitor Nutlin-3a acted synergistically in p53 wild-type cells. Interestingly, GSK2830371 sensitized MCL cells to bortezomib and doxorubicin in p53 wild-type and mutant cells; p38 signaling appeared to be involved in the GSK2830371/bortezomib lethality. PPM1D inhibition may represent a novel therapeutic strategy for MCL, which can be exploited in combination therapeutic strategies for MCL.

## INTRODUCTION

Mantle cell lymphoma (MCL) is a distinct type of non-Hodgkin lymphoma genetically characterized by balanced t(11:14) translocation and cyclin D1 overexpression [1, 2]. Despite recent improvements in therapy, the great majority of patients with MCL remain incurable [3–6]. The selection of chemoresistant lymphoma subclones is thought to occur after conventional chemotherapies and targeted therapies, leading to an inevitable relapse of MCL [7].

Many chemotherapeutic agents currently used for the treatment of MCL induce DNA damage and activate the tumor suppressor p53 [8]. Mutations in p53 are relatively rare in MCL (approximately 15% of cases). However, DNA damaging agents could potentially accelerate clonal evolution by inducing new mutations in the original clones. Recent preclinical studies have found that the reactivation of p53 consequent to inhibition of the MDM2-p53 interaction or XPO1 (a nuclear exporter for p53) via non-genotoxic small molecule inhibitors can induce apoptosis in lymphoma cells [9–11]. Furthermore, data from early phase clinical trials have demonstrated encouraging anti-tumor activities of MDM2 and XPO1 inhibitors in lymphoma patients [12, 13]. One limitation of single-agent p53 activation therapies is that a sufficient p53 response has not been attained in some cancer cells with wild-type p53. In MCL, p53 stabilization has not always led to p53-mediated transcriptional activation [11]. In this context, a more potent reactivation of wild-type p53 using a rational combination of non-genotoxic small molecules would constitute a novel strategy [14, 15]. On the other hand, the possibility of selecting p53-mutant sub-clones remains a potential clinical concern regarding the use of p53 inducers [16, 17]. Considering the intratumoral heterogeneity and subclonal architecture of MCL [18], a p53-activating targeted therapy would ideally possess p53-independent anti-tumor activities.

PPM1D (protein phosphatase, Mg2+/Mn2+ dependent 1D), also known as WIP1 (wild-type p53-induced phosphatase 1), is a serine/threonine phosphatase that negatively regulates key DNA damage response proteins, including p53, p38 MAPK, histone H2A.X, and ATM [19] and exhibits an oncogenic activity [20]. PPM1D overexpression or amplification has been reported in various cancers, including lung, breast, kidney, and ovarian cancers [21–24]. GSK2830371, a novel PPM1D inhibitor, binds to a flap subdomain and, thus, regulates the enzymatic and substrate recognition activities of PPM1D [25]. Treatment of cancer cells with GSK2830371 has been found to increase the phosphorylation of PPM1D substrates, especially phosphorylated p53 at serine 15, and to induce growth inhibition and apoptosis in the cells harboring wild-type p53 [25–28]. GSK2830371 has not been studied in MCL.

In this study, we investigated the clinical significance of PPM1D and the anti-lymphoma effects of GSK2830371 in MCL.

## RESULTS

### PPM1D overexpression is associated with a high proliferation-related gene expression signature and poor disease prognosis in patients with MCL

**Figure 1:**
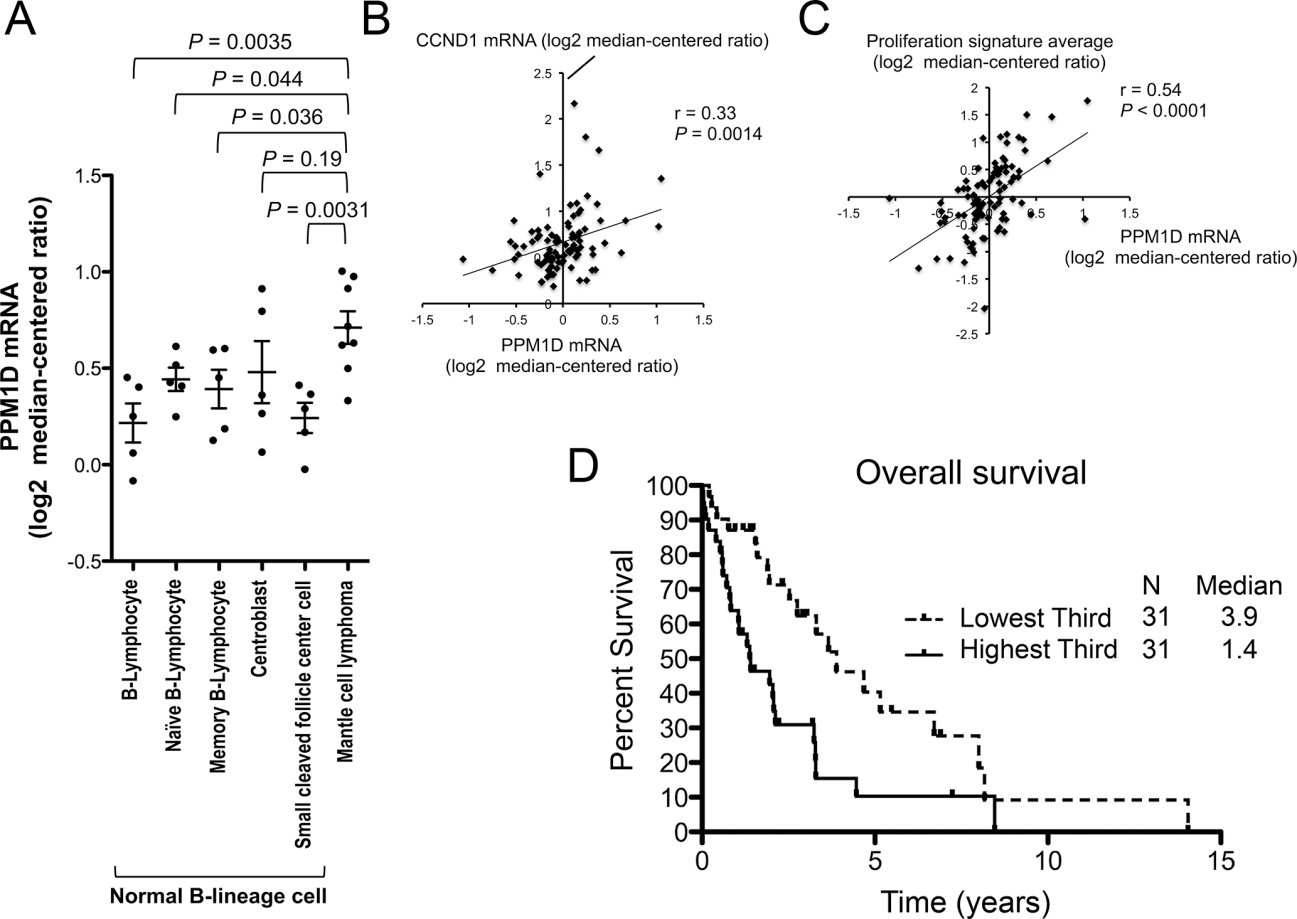
High PPM1D expression is associated with a highly proliferative disease phenotype and poor prognosis in patients with mantle cell lymphoma (MCL) (**A**) PPM1D mRNA levels in normal B-lineage cells at different stages of maturation and MCL cells. (**B**) Positive correlation of PPM1D mRNA levels with CCND1 mRNA levels. (**C**) Positive correlation of PPM1D mRNA levels with proliferation signature averages. (**D**) Kaplan–Meier plots of the prognostic relevance of PPM1D mRNA expression on overall survival in patients with MCL.

PPM1D mRNA expression levels in samples from patients with MCL were determined using Oncomine data (Compendia Bioscience, Ann Arbor, MI, USA). Our gene expression analyses revealed an increase in PPM1D mRNA expression in MCL samples (*n* = 8) relative to normal naïve B lymphocytes (*n* = 5; *P* = 0.044; GSE2350 [29]), which are thought to be a normal counterpart of MCL cells (Figure 1A). The levels in MCL patient samples were significantly higher than those in four of five normal B-lineage cell types at different stages of maturation (Figure 1A). PPM1D mRNA levels positively correlated with CCND1 (Cyclin D1) mRNA levels (*r* = 0.33, *P* = 0.0014; *n* = 92; Figure 1B) and with proliferation signature averages (*r* = 0.54, *P* < 0.0001; *n* = 92; Figure 1C) in a series of MCL samples (http://llmpp.nih.gov/MCL [30]). The proliferation signature has been shown to be a quantitative integrator of oncogenic events and survival predictor in MCL [30]. Importantly, increased PPM1D expression at diagnosis was itself associated with a poorer prognosis in MCL patients (median overall survival of 3.9 years and 1.4 years for cases in the lowest and highest PPM1D expression tertiles, respectively; *P* = 0.0047; Bonferroni-corrected threshold 0.0167; Figure 1D). The median overall survival of the middle expression tertile was 3.1 years, representing an intermediate value between those of highest and lowest tertiles. These results indicate that PPM1D overexpression is associated with a highly proliferative disease phenotype and poor prognosis in patients with MCL and that PPM1D may be a potential therapeutic target in MCL. PPM1D mRNA levels were compared across major lymphoma types (GSE2350 [29]). The levels in MCL were as high as those in aggressive lymphomas including Burkitt's lymphoma and diffuse large B-cell lymphoma, and were significantly higher than those in indolent lymphomas including chronic lymphocytic leukemia/small lymphocytic lymphoma (*P* = 0.0076) and follicular lymphoma (*P* = 0.011) (Supplementary Figure S1). PPM1D expression was also determined at the protein level and, in accordance with mRNA expression results, the levels were higher in MCL cells than normal lymphocytes (Supplementary Figure S2).

### GSK2830371 exerts anti-proliferative and apoptotic effects on MCL cells in a partially p53-dependent manner

**Figure 2:**
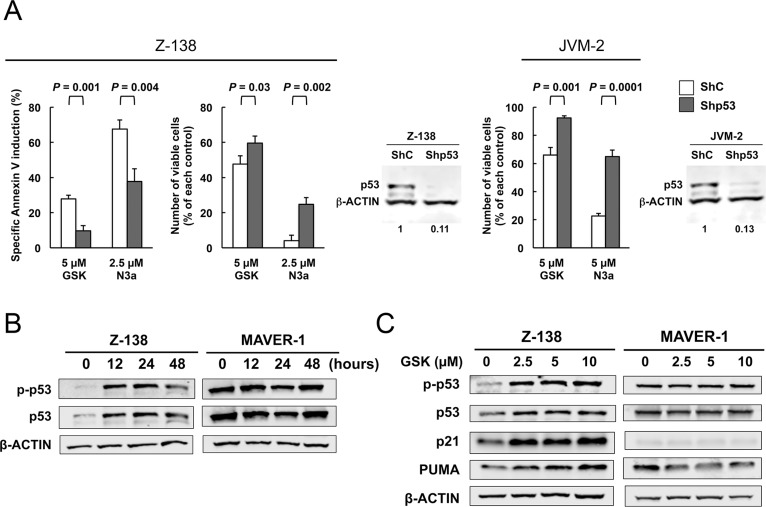
The PPM1D inhibitor GSK2830371 induces cell cycle arrest and apoptosis in mantle cell lymphoma (MCL) cells partially in a p53-dependent manner (**A**) Wild-type p53 MCL cells transduced with lentivirus encoding either control shRNA (ShC) or p53-specific shRNA (Shp53) were incubated with the indicated concentrations of GSK2830371 (GSK) or Nutlin-3a (N3a), and the numbers of viable cells and annexin V-positive fractions were determined. (**B**) Expression of total and phosphorylated p53 proteins in Z-138 and MAVER-1 cells treated with 10 μM GSK2830371. (**C**) Expression of p53-related proteins in Z-138 and MAVER-1 cells after a 12-hour treatment with the indicated concentrations of GSK2830371. β-actin was used as loading control. Results are representative of three independent experiments.

We next examined the effect of the PPM1D inhibitor GSK2830371 on cell growth and viability in MCL cell lines. Cells were treated with various concentrations of GSK2830371 (0, 2.5, 5, 10, or 20 μM) for 72 hours, and subjected to evaluations of IC50 values (inhibitory concentration at which cell growth is inhibited by 50% as determined by trypan blue dye exclusion assay) and ED50 values (effective concentration inducing 50% killing as measured by annexin V positivity) at 48 and 72 hours (Table 1). Z-138, JVM-2, and Granta-519 express wild-type p53, whereas MINO, Jeko-1, REC-1, MAVER-1, and NCEB-1 express mutant p53. GSK2830371 exerted dose-dependent anti-proliferative and/or apoptotic effects on sensitive MCL cells at concentrations ranging from 2.5 to 10 μM, although these effects were modest in most cell lines except for Z-138. The highest concentration of GSK2830371 (20 μM) did not exert stronger anti-proliferative or apoptotic effects relative to a concentration of 10 μM. Notably, 10 μM GSK2830371 inhibited the growth of p53 wild-type Z-138, JVM-2, and Granta-519 cells by 68%, 38%, and 39% at 48 hours, respectively (Table 1). The anti-proliferative effects on p53 mutant cells ranged from 7% to 32%, which were significantly lower than those observed in p53 wild-type cells (*P* = 0.036). GSK2830371 induced ≥ 50% killing only in Z-138 cells. In sensitive Z-138 cells, GSK2830371 caused a significant loss of mitochondrial membrane potential in addition to annexin V induction (16.7 ± 2.9% specific loss after 72-hour treatment of 5 μM GSK2830371), confirming its apoptotic activity. To further investigate whether the p53 status determines GSK2830371 sensitivity, p53 wild-type Z-138 and JVM-2 cells were transduced with lentiviruses encoding either negative control shRNA (ShC) or p53-specific shRNA (Shp53) to generate stable shRNA-expressing cells. p53-specific shRNA reduced p53 levels by > 85% (Figure 2A). The isogenic cells were exposed for 72 hours to GSK2830371 or the selective MDM2 inhibitor Nutlin-3a, which activates p53. Nutlin-3a was included to determine the functional knockdown efficiency of p53. As shown in Figure 2A, p53 knockdown Z-138 cells were less sensitive to GSK2830371- and Nutlin-3a-induced growth inhibition and apoptosis than were control cells, suggesting that GSK2830371 activates p53-mediated signaling to induce growth inhibition and apoptosis. Similarly, p53 knockdown desensitized JVM-2 cells to GSK2830371-induced growth inhibition (Figure 2A). Taken together, our data suggest that the anti-MCL effects of GSK2830371 are partially p53-dependent.

**Table 1:** Anti-proliferative and apoptotic effects of GSK2830371 in mantle cell lymphoma (MCL) cell lines

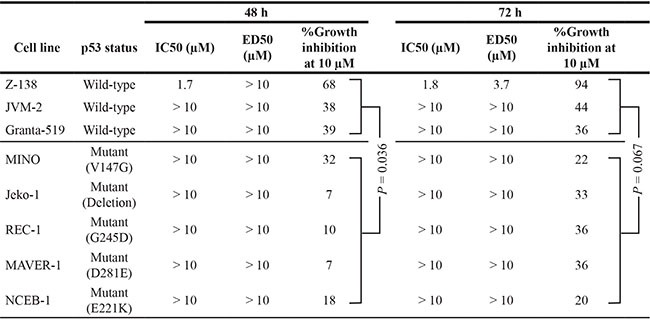

IC50, inhibitory concentration at which cell growth is inhibited by 50% as determined by trypan blue dye exclusion assay; ED50, effective concentration inducing 50% killing as measured by annexin V positivity.

### GSK2830371 increases the levels of total and phosphorylated p53 and activates wild-type p53 signaling in MCL cells

To investigate whether GSK2830371 activates wild-type p53 signaling, the cells most sensitive and least sensitive to this inhibitor, Z-138 cells expressing wild-type p53 (ED50 of 3.7 μM after a 72-hour exposure to 10 μM GSK2830371) and MAVER-1 cells expressing mutant p53 (< 1% annexin V induction after 72 hours), respectively, were exposed to GSK2830371, and the levels of total and phosphorylated p53 in these cells were determined. As shown in Figure 2B, increases in both total and phosphorylated p53 levels were detected in Z-138 cells as early as 12 hours after exposure. Decreased levels of p53 at 48 hours relative to those at 12 and 24 hours appeared to reflect decreased viability in GSK2830371-sensitive Z-138 cells. MAVER-1 cells expressed high basal levels of total and phosphorylated p53, which is common in cells that express nonfunctional mutant p53, and these levels did not change significantly after treatment. GSK2830371 also induced the expression of transcriptional targets of wild-type p53. As shown in Figure 2C, GSK2830371 induced p21 and PUMA expression in Z-138 cells. p21 and PUMA play major roles in p53-mediated cell cycle arrest and apoptosis, respectively [31]. GSK2830371 treatment did not affect the p21 and PUMA levels in MAVER-1 cells. These data suggest that GSK2830371 increases the levels of total and phosphorylated p53 and activates wild-type p53 signaling.

### PPM1D expression levels per se and the phosphorylation statuses of PPM1D targets were not determinants of MCL cell sensitivity to GSK2830371

**Figure 3:**
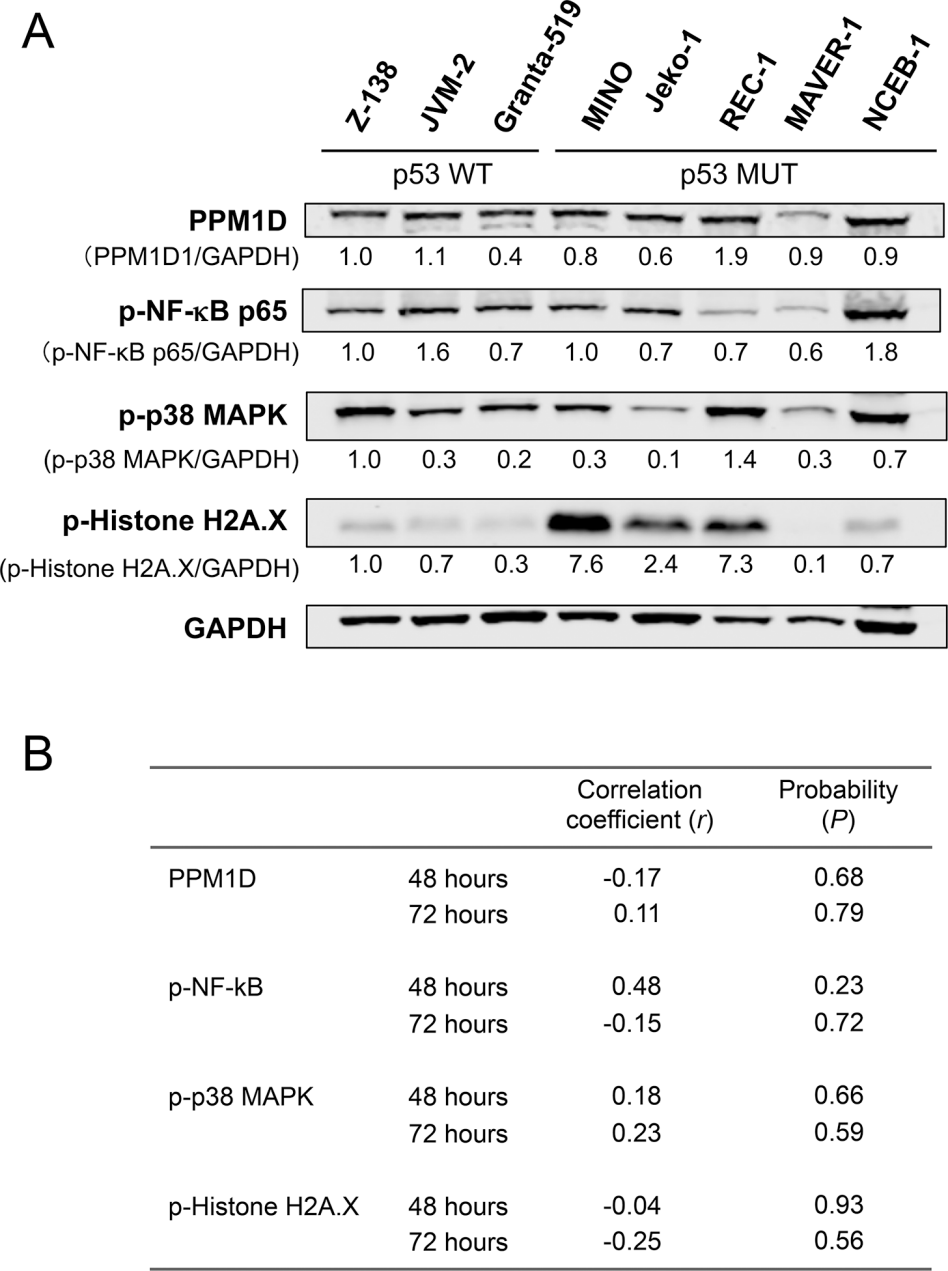
Basal levels of PPM1D and phosphorylated protein levels of PPM1D targets did not determine the sensitivity of mantle cell lymphoma (MCL) cells to GSK2830371 (**A**) Basal expression levels of PPM1D and its target proteins in MCL cell lines. (**B**) Correlation coefficient and probability values of the 48-hour and 72-hour growth inhibitory effects of 10 μM GSK2830371 relative to PPM1D-related proteins. WT, wild-type; MUT, mutant.

PPM1D is a serine/threonine phosphatase that targets multiple proteins. We investigated whether basal PPM1D levels or the phosphorylation statuses of PPM1D targets were associated with MCL cell sensitivity to GSK2830371. The levels of PPM1D and phosphorylated NFkB-p65, p38 MAPK, and histone H2A.X varied widely among MCL cell lines (Figure 3A). None of these levels correlated with cell sensitivity to GSK2830371 (Figure 3B). Induced levels of phosphorylated NFkB-p65, p38 MAPK and histone H2A.X by 24-hour treatment with 10 μM GSK2830371 were also correlated with the sensitivity. Again, no statistically significant correlation was observed (Supplementary Figure S3).

### GSK2830371 potentiates the anti-lymphoma effects of Nutlin-3a, bortezomib, and doxorubicin

**Figure 4:**
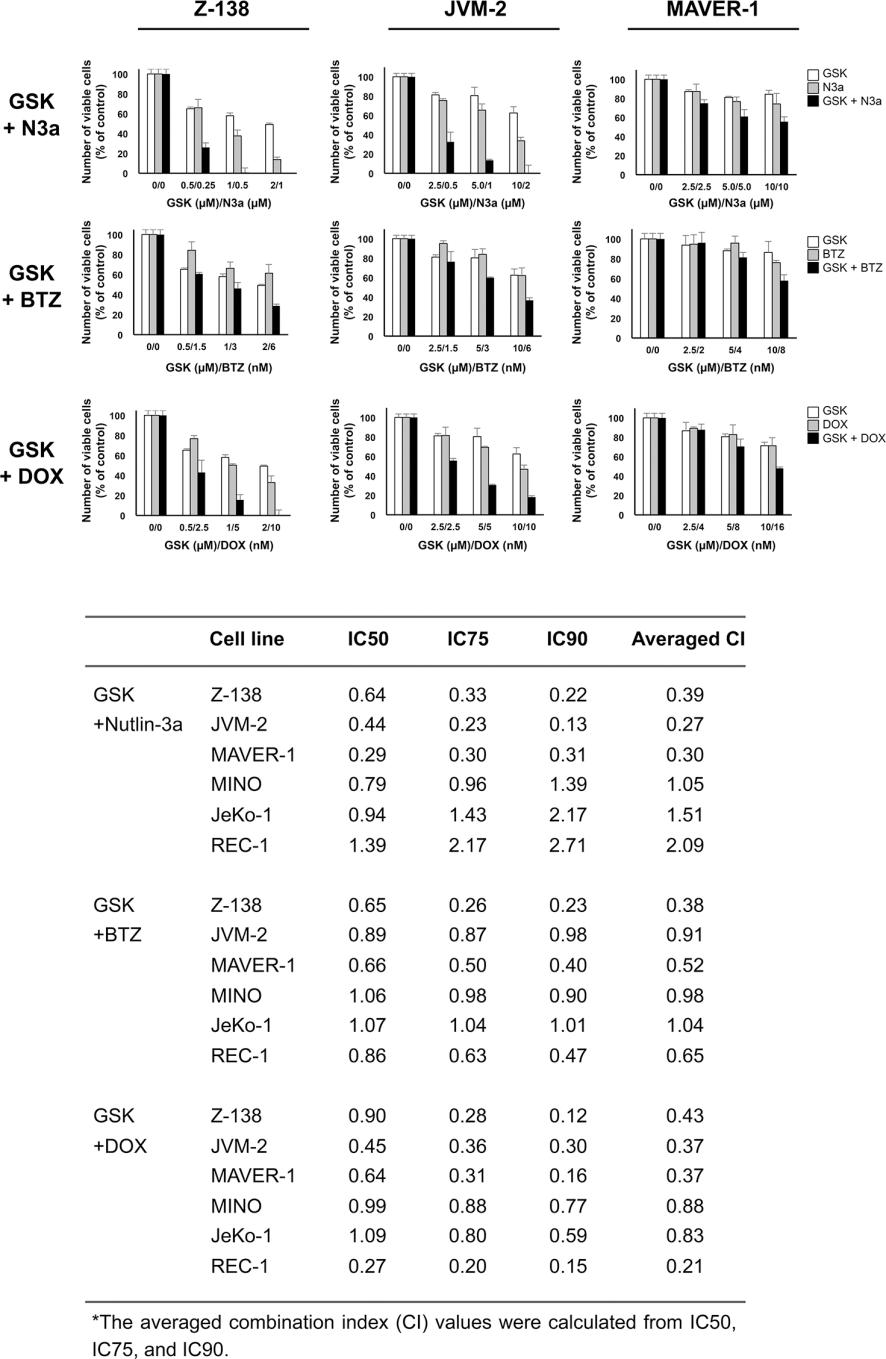
GSK2830371 potentiates the growth inhibitory effects of Nutlin-3a, bortezomib, and doxorubicin Mantle cell lymphoma (MCL) cells were treated for 72 hours with GSK2830371 (GSK) and Nutlin-3a (N3a), bortezomib (BTZ), or doxorubicin (DOX) either as individual agents or in combination, after which the numbers of viable cells were determined. The combination index (CI) values for the anti-proliferative effects were calculated for each combination.

**Figure 5:**
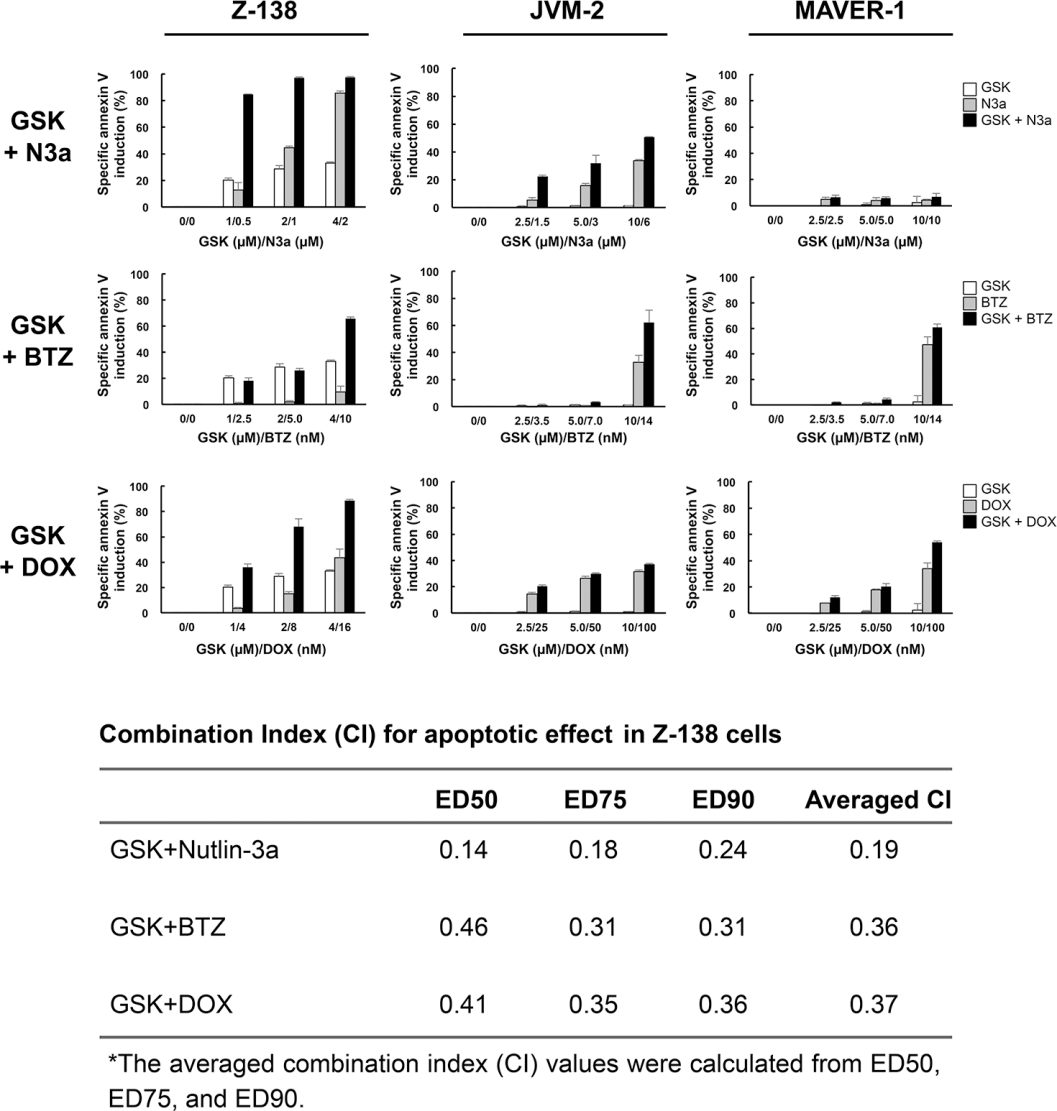
GSK2830371 potentiates the apoptotic effects of Nutlin-3a, bortezomib, and doxorubicin Mantle cell lymphoma (MCL) cells were treated for 72 hours with GSK2830371 (GSK) and Nutlin-3a (N3a), bortezomib (BTZ), or doxorubicin (DOX) either as individual agents or in combination, after which the annexin V-positive fractions were determined. The combination index (CI) values for the apoptotic effect were calculated in Z-138 cells for each combination.

We investigated whether GSK2830371-mediated PPM1D inhibition would enhance the anti-lymphoma effects of other compounds. Cells were treated with GSK2830371 and Nutlin-3a, bortezomib, or doxorubicin at 1/4 IC50, 1/2 IC50, or IC50 concentrations (1/4 ED50, 1/2 ED50, or ED50 concentrations in sensitive Z-138 cells) for 72 hours, after which the numbers of viable cells and the annexin V-positive fractions were determined. First, we combined GSK2830371 with Nutlin-3a. The selective MDM2 inhibitor Nutlin-3a activates wild-type p53 signaling, inhibits cell growth, and induces apoptosis in some types of cancer cells, including MCL cells [9, 32]. As shown in Figures 4 and 5, GSK2830371 enhanced Nutlin-3a-induced growth inhibition and apoptosis induction in p53 wild-type Z-138 and JVM-2 cells. Some synergism was observed between GSK2830371 and Nutlin-3a with respect to growth inhibition in p53 mutant MAVER-1 cells, although the inhibitory effect was modest even in the combination group (approximately 40% growth inhibition) (Figure 4). Since Nutlin-3a has been shown to induce p73 (a p53 homolog) by inhibiting MDM2 in MCL cells [9], and thus the observed synergy might be attributable to the phosphorylation of PPM1D targets and activation of MDM2 targets other than p53. The GSK2830371/Nutlin-3a combination did not induce significant apoptosis in MAVER-1 cells (Figure 5), implying that the dual inhibition of PPM1D and MDM2 is not sufficient to induce cell death in p53-defective cells. The proteasome inhibitor bortezomib and conventional chemotherapeutic agent doxorubicin have been used for the treatment of MCL [1–3]. The results of combination studies involving GSK2830371 with bortezomib or doxorubicin suggested that GSK2830371 enhances bortezomib- and doxorubicin-induced growth inhibition and apoptosis, irrespective of the p53 mutational status (Figures 4 and 5). We investigated the potentiating effect in three additional MCL cell lines (MINO, JeKo-1 and REC-1) that express mutant p53 and are resistant to GSK2830371-induced apoptosis. The potentiation was prominently observed in REC-1 cells (Figures 4 and Supplementary Figure S4), implying that the potentiation effect of GSK2830371 depends on p53-unrelated intrinsic abnormalities in MCL cells. The p53-independent potentiation effect of GSK2830371 on the anti-lymphoma effects of bortezomib and doxorubicin supports the idea that PPM1D inhibition could be widely exploited in combination therapeutic strategies for MCL.

### GSK2830371 enhances p53 activation and apoptosis induction mediated by Nutlin-3a, bortezomib, and doxorubicin

**Figure 6:**
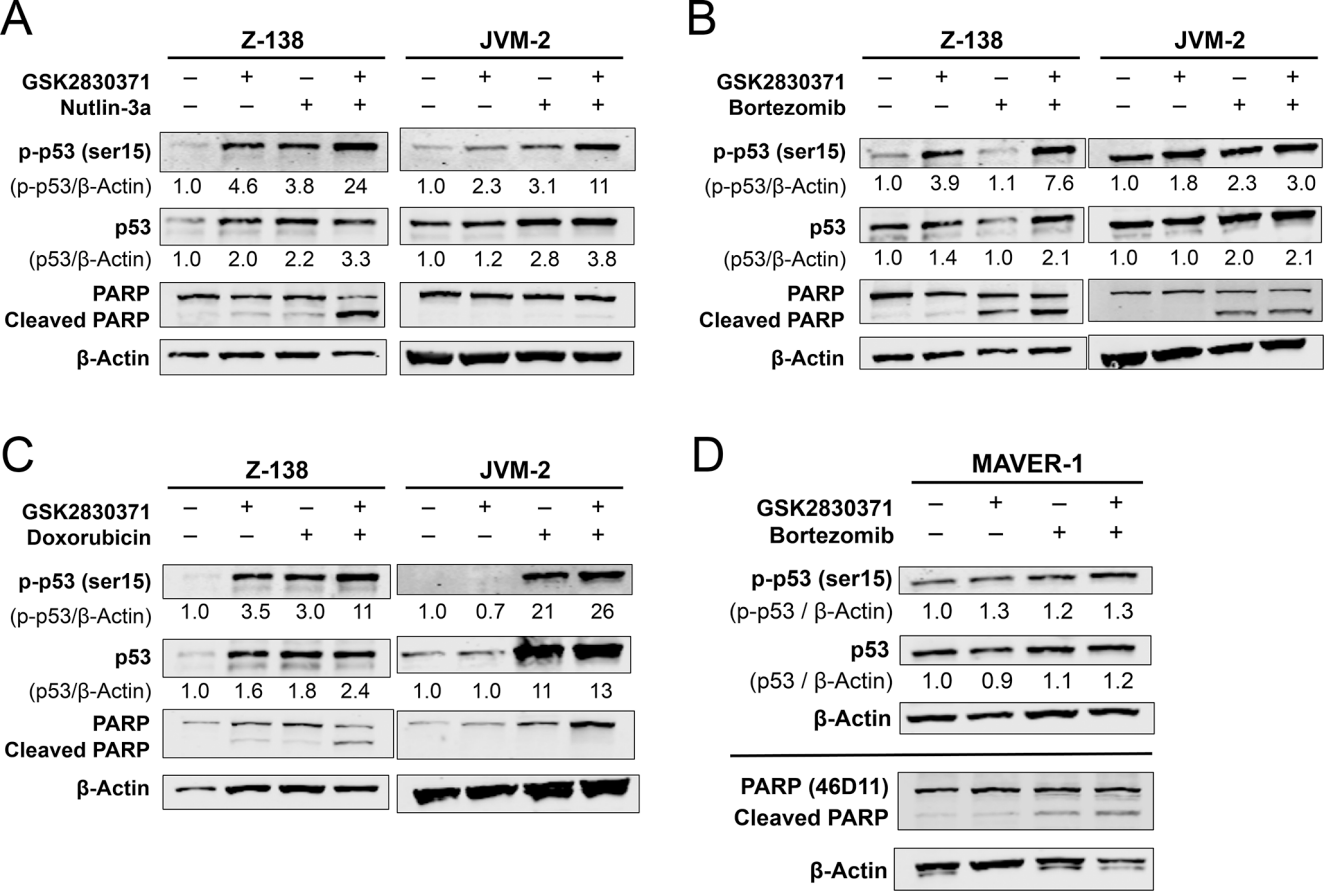
GSK2830371 enhances p53 activation and apoptosis induction by Nutlin-3a, bortezomib, and doxorubicin Z-138 and JVM-2 cells were treated for 24 hours with GSK2830371 (4 μM for Z-138 and 10 μM for JVM-2) or (**A**) Nutlin-3a (2 μM for Z-138 and 6 μM JVM-2), (**B**) bortezomib (12 nM for Z-138 and 14 nM for JVM-2), or (**C**) doxorubicin (16 nM Z-138 and 100 nM JVM-2) either as individual agents or in combination, after which the levels of total and phosphorylated p53 and PARP were determined. (**D**) MAVER-1 cells were treated with 10 μM GSK2830371 and 14 nM bortezomib either as individual agents or in combination. The expression levels of total and phosphorylated p53 were determined after 24 hours of treatment; the degree of PARP cleavage was determined after 48 hours. The intensities of immunoblot signals were quantified, the intensities relative to β-actin were calculated, and the levels in untreated cells were set at 1.0. The results are representative of three independent experiments.

To investigate whether GSK2830371 could enhance p53 activation and cell death induced by Nutlin-3a, bortezomib, or doxorubicin, the expression of total and phosphorylated p53 and degree of PARP cleavage were determined in Z-138 and JVM-2 cells after a 24-hour exposure to GSK2830371 with Nutlin-3a (Figure 6A), bortezomib (Figure 6B), or doxorubicin (Figure 6C). All combinations significantly increased the levels of total and phosphorylated p53 in both cells and induced a higher degree of PARP cleavage in Z-138 cells. The induction of p53 was most prominent in cells treated with the GSK2830371/Nutlin-3a combination. Bortezomib has a distinct drug target, the proteasome, and has been shown to induce apoptosis through both p53-dependent and -independent pathways. Based on the observed synergism between GSK2830371 and bortezomib (Figures 4, 5 and Supplementary Figure S4), we determined the levels of total and phosphorylated p53 and cleaved PARP in p53-defective MAVER-1 cells exposed to GSK2830371 and bortezomib, both as individual agents and in combination. The p53 levels did not change significantly even in cells treated with the drug combination (Figure 6D). Nevertheless, the GSK2830371/bortezomib combination induced a higher degree of PARP cleavage relative to individual agents, suggestive of p53-independent activity.

### GSK2830371 sensitizes p53 mutant MCL cells to bortezomib possibly through p38 MAPK activation in addition to p53 activation

**Figure 7:**
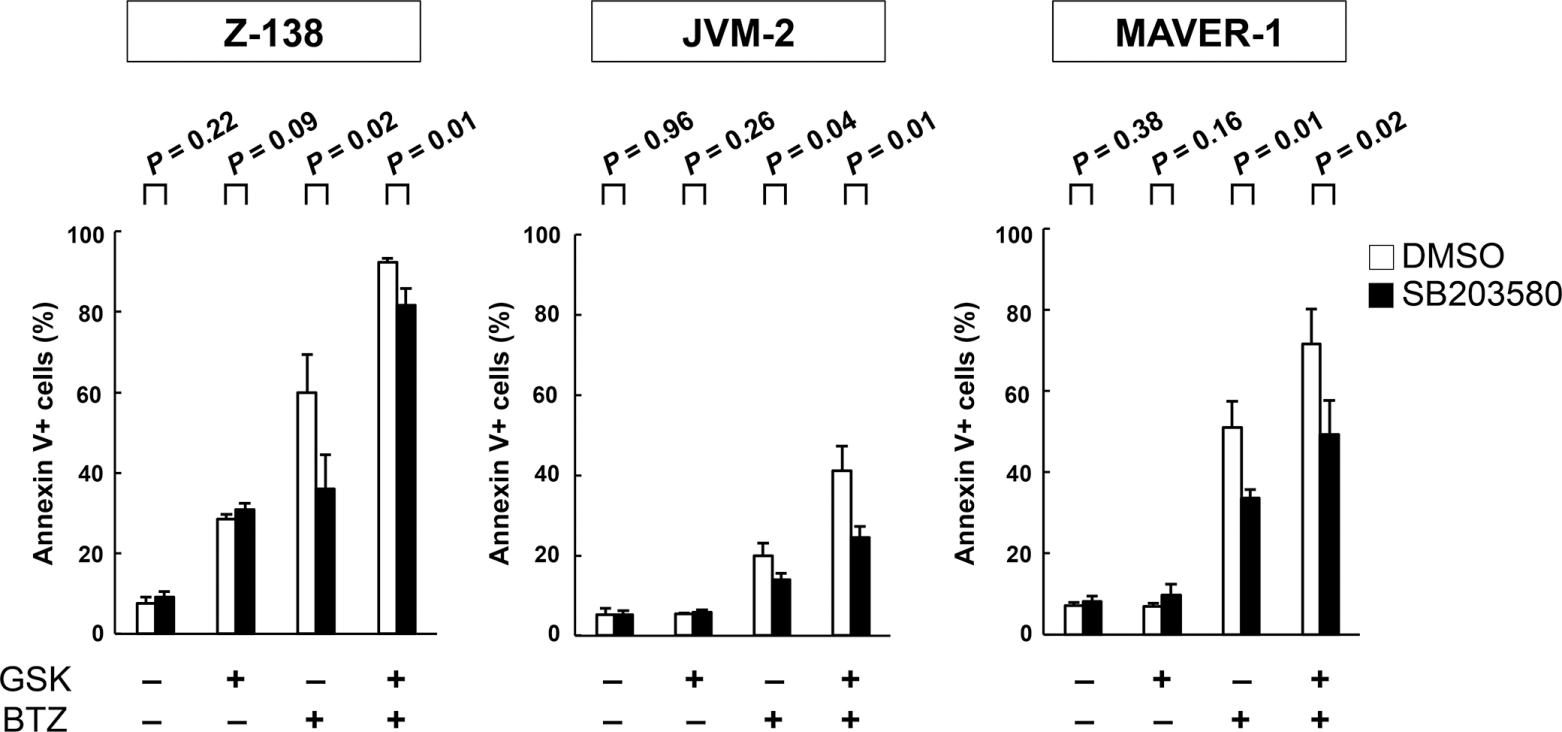
The selective p38 inhibitor SB203580 attenuates bortezomib- and GSK2830371/bortezomib-induced lethality Z-138, JVM-2, and MAVER-1 cells were exposed to GSK2830371 and bortezomib in the presence or absence of the selective p38 inhibitor SB203580; the annexin V-positive fractions were determined after 72 hours. SB203580 was added to the cells 30 minutes before the administration of GSK2830371 or bortezomib.

Both GSK2830371 and bortezomib target the p38 MAPK signaling pathway [33–35], and activation of p38 MAPK has been found to cause MCL cell death [35]. As shown in Supplementary Figure S5, GSK2830371 and bortezomib synergistically increased levels of phosphorylated p38 MAPK in a p53-independent manner. To investigate whether p38 MAPK activation plays a significant functional role in GSK2830371/bortezomib-mediated lethality in MCL cells, Z-138, JVM-2, MAVER-1 and REC-1 cells were exposed to GSK2830371 and bortezomib, either as individual agents or in combination, in the presence or absence of the selective p38 inhibitor SB203580 at 10 μM; annexin V-positive fractions were subsequently analyzed. SB203580 has been found to be one of the most specific p38 MAPK inhibitors [36]. SB203580 did not influence sensitivity to GSK2830371 (Figure 7). Interestingly, SB203580 significantly attenuated bortezomib- and GSK2830371/bortezomib-induced lethality in three out of four cell lines (Figure 7). The protective effect of SB203580 from GSK2830371/bortezomib-induced lethality was lesser in Z-138 cells relative to JVM-2 and MAVER-1 cells, and this difference might reflect a key role of p53-mediated apoptosis in the former cell type. These data raise the possibility that GSK2830371 sensitizes MCL cells to bortezomib partially through p38 MAPK activation. SB203580 did not protect MCL cell from doxorubicin- or GSK2830371/doxorubicin-induced lethality.

## DISCUSSION

In this study, we clarified the pathophysiological significance of PPM1D and therapeutic targeting of this protein by the novel PPM1D inhibitor GSK2830371 in MCL. Patient-derived MCL cells expressed higher levels of PPM1D mRNA than normal B-cell counterpart cells. Moreover, higher PPM1D mRNA expression was clinically associated with a more aggressive clinical phenotype and poorer disease prognosis. These data suggest that dysregulated PPM1D is actively involved in the pathogenesis of MCL and that PPM1D is a potential therapeutic target for MCL. However, the investigated cell lines were relatively insensitive to single-agent GSK2830371. The modest anti-cancer activity as a single agent has been recently described in solid cancer cells [25, 26]. GSK2830371 has the advantageous ability to induce the phosphorylation of p53 at Ser15 in a non-genotoxic manner. We, therefore, hypothesized that GSK2830371 could potentiate the anti-lymphoma effects of p53 inducers in MCL cells and confirmed this hypothesis using combination studies in which cells were treated with GSK2830371 and the MDM2 inhibitor Nutlin-3a, the proteasome inhibitor bortezomib, and the anthracycline doxorubicin.

Clinical trials have revealed single-agent clinical activity of MDM2 inhibitors in patients with p53 wild-type tumors [12, 37]. MDM2 inhibitors potently activate wild-type p53 signaling and, as expected, the p53 genetic status is a major determinant of patient responses to MDM2 inhibitors. Nevertheless, the sensitivity of wild-type p53 tumors to MDM2 inhibitors has varied widely among cancer types and patients. This highly divergent sensitivity has also been reported in p53 wild-type cell lines and patient-derived samples, and is likely associated with a range of genetic variables that may limit p53 reactivation or modulate cell fates after p53 reactivation [38]. In MCL, a limited activity of p53-mediated transcription has been reported [11]. We found that GSK2830371 potentiated the responses of MCL cells to the MDM2 inhibitor Nutlin-3a in association with a profound increase in phosphorylated p53 levels. The phosphorylation of p53 at Ser15 increases p53 transcriptional activity [31]. Esfandiari et al. recently reported a similar finding [26], in which GSK2830371 potentiated sensitivities to MDM2 inhibitors in p53 wild-type cancer cell lines harboring either PPM1D copy number gains/elevated expression or gain-of-function truncating mutations. Other researchers have also reported an association between higher PPM1D expression levels and increased sensitivity to GSK2830371 [27, 28]. As our data demonstrated increased PPM1D expression in MCL cells, dual inhibition of PPM1D and MDM2 would be a logical approach to the treatment of MCL and, particularly, the aggressive form of this disease.

GSK2830371 potentiated the anti-lymphoma effects of bortezomib and doxorubicin in p53 wild-type and mutant MCL cells. Previous studies have shown that the anti-cancer effects of GSK2830371 are observed exclusively in p53 wild-type cells [25–28]. For example, Richter et al. have reported that p53 mutations predict GSK2830371 resistance in neuroblastoma cell lines [27]. However, PPM1D is not a specific p53 phosphatase but instead targets a wide range of proteins. Most PPM1D substrates either initiate or contribute to cellular stress signals and the removal of activating phosphorylation has been thought to liberate cells from cellular stress [39]. p38 MAPK is generally a pro-apoptotic and stress-related protein that is regulated by PPM1D and the proteasome [33–35] and its activation has been found to cause MCL cell death [35]. Our data, which indicate that the pharmacologic inhibition of p38 significantly reduced GSK2830371/bortezomib lethality in both p53 wild-type and mutated MCL cells, suggest that this stress pathway contributes functionally to apoptosis. The p53-independent potentiation effect was also observed with the combination of GSK2830371 and doxorubicin, although doxorubicin exerts its anti-tumor activity through a number of proposed mechanisms of action, and thus, the mechanism relevant to this study could not be elucidated. Although a single-agent activity of GSK2830371 was modest against MCL cells, especially against those with mutant p53, its p53-independent potentiation effect on bortezomib and doxorubicin may support the use of PPM1D inhibitors as part of a combination therapeutic strategy for MCL.

## MATERIALS AND METHODS

### Reagents

GSK2830371 was purchased from Tocris Bioscience (Bristol, UK), and the MDM2 inhibitor Nutlin-3a and p38 MAPK inhibitor SB203580 were obtained from Cayman Chemical Company (Ann Arbor, MI, USA).

### Cells and cell culture

A total of 8 MCL cell lines were cultured in RPMI 1640 medium containing 20% heat-inactivated fetal bovine serum (Table 1). Z-138, JVM-2, MINO, JeKo-1, REC-1, MAVER-1, and NCEB-1 were purchased from ATCC (Rockville, MD) and Granta-519 from DSMZ (Braunschweig, Germany). Z-138, JVM-2, and Granta-519 cells express wild-type p53, whereas MINO, JeKo-1, REC-1, MAVER-1, and NCEB-1 cells express mutant p53 [11]. Z-138 and JVM-2 cells were transduced with retroviruses encoding either p53-specific shRNA or scrambled shRNA to generate stable shRNA-expressing cells [11]. MCL cell lines were harvested during log-phase growth, seeded at a density of 2 × 10^5^ cells/ml, and treated with compounds as indicated. The number of viable cells was evaluated through triplicate counts of trypan blue dye-excluding cells. Patient samples were analyzed under the protocol approved by the institutional review board at Saga University (2014-10-05). Heparinized peripheral blood samples were obtained according to institutional guidelines per the Declaration of Helsinki.

### Apoptosis analysis

Flow-cytometric determination of Annexin V binding and mitochondrial membrane potential loss was performed [10]. Apoptosis was quantified as the percentage of annexin V-positive cells. The frequency of drug-specific apoptosis was assessed using the formula (% test–% control) × 100/(100–% control).

### Western blot analysis

Western blot analysis was performed as described previously [11]. The following antibodies were used: anti-PPM1D (sc-20712; Santa Cruz Biotechnology, Dallas, TX), anti-phospho-p53 (Ser15) (#9284; Cell Signaling Technology, Beverly, MA); anti-p53 (DO-1; Santa Cruz Biotechnology); anti-p21 (EA10; EMD Biosciences, San Diego, CA); anti-PUMA (ab9643; Abcam, Cambridge, MA); anti-phospho-NFκB-p65 (Ser536) (#3033; Cell Signaling Technology); anti-phospho-p38 MAPK (Thr180/Tyr182) (#9211; Cell Signaling Technology); anti-phospho-histone H2A.X (Ser139) (#2577; Cell Signaling Technology); anti-PARP (#9532; Cell Signaling Technologies); anti-GAPDH (#2118; Cell Signaling Technologies); and anti-β-actin (13E5; Sigma Chemical Co., St Louis, MO).

### Statistical analyses

Statistical analyses were performed using a two-sided Student's *t*-test, the non-parametric Mann–Whitney *U*-test, and the Pearson correlation coefficient as appropriate. Kaplan–Meier curves were used in combination with the log-rank test for survival analyses. Bonferroni correction was applied when multiple comparisons were performed. A *P* value < 0.05 was considered statistically significant. Average values were expressed as means ± standard deviations (SD). The combination index (CI), a numerical description of combination effects, was calculated using the more stringent statistical assumption of mutually nonexclusive modes of action. By this method, CI values indicate the following: < 0.3, strong synergism; 0.3–0.7, synergism; 0.7–0.85, moderate synergism; 0.85–0.9, slight synergism; 0.9–1.1, nearly additive; 1.1–1.2, slight antagonism; 1.2–1.45, moderate antagonism; 1.45–3.3, antagonism; and > 3.3, strong antagonism [40].

## SUPPLEMENTARY MATERIALS



## References

[1] Cheah CY, Seymour JF, Wang ML (2016). Mantle Cell Lymphoma. J Clin Oncol.

[2] Jares P, Campo E (2008). Advances in the understanding of mantle cell lymphoma. Br J Haematol.

[3] Robak T, Huang H, Jin J, Zhu J, Liu T, Samoilova O, Pylypenko H, Verhoef G, Siritanaratkul N, Osmanov E, Alexeeva J, Pereira J, Drach J (2015). Bortezomib-based therapy for newly diagnosed mantle-cell lymphoma. N Engl J Med.

[4] Rummel M, Kaiser U, Balser C, Stauch M, Brugger W, Welslau M, Niederle N, Losem C, Boeck HP, Weidmann E, von Gruenhagen U, Mueller L, Sandherr M (2016). Bendamustine plus rituximab versus fludarabine plus rituximab for patients with relapsed indolent and mantle-cell lymphomas: a multicentre, randomised, open-label, non-inferiority phase 3 trial. Lancet Oncol.

[5] Wang ML, Rule S, Martin P, Goy A, Auer R, Kahl BS, Jurczak W, Advani RH, Romaguera JE, Williams ME, Barrientos JC, Chmielowska E, Radford J (2013). Targeting BTK with ibrutinib in relapsed or refractory mantle-cell lymphoma. N Engl J Med.

[6] Dunleavy K (2016). Immunomodulatory agents in mantle cell lymphoma. Lancet Oncol.

[7] Martin P, Maddocks K, Leonard JP, Ruan J, Goy A, Wagner-Johnston N, Rule S, Advani R, Iberri D, Phillips T, Spurgeon S, Kozin E, Noto K (2016). Post-ibrutinib outcomes in patients with mantle cell lymphoma. Blood.

[8] Delfau-Larue MH, Klapper W, Berger F, Jardin F, Briere J, Salles G, Casasnovas O, Feugier P, Haioun C, Ribrag V, Thieblemont C, Unterhalt M, Dreyling M (2015). High-dose cytarabine does not overcome the adverse prognostic value of CDKN2A and TP53 deletions in mantle cell lymphoma. Blood.

[9] Tabe Y, Sebasigari D, Jin L, Rudelius M, Davies-Hill T, Miyake K, Miida T, Pittaluga S, Raffeld M (2009). MDM2 antagonist nutlin-3 displays antiproliferative and proapoptotic activity in mantle cell lymphoma. Clin Cancer Res.

[10] Kojima K, Konopleva M, McQueen T, O'Brien S, Plunkett W, Andreeff M (2006). Mdm2 inhibitor Nutlin-3a induces p53-mediated apoptosis by transcription-dependent and transcription-independent mechanisms and may overcome Atm-mediated resistance to fludarabine in chronic lymphocytic leukemia. Blood.

[11] Yoshimura M, Ishizawa J, Ruvolo V, Dilip A, Quintás-Cardama A, McDonnell TJ, Neelapu SS, Kwak LW, Shacham S, Kauffman M, Tabe Y, Yokoo M, Kimura S (2014). Induction of p53-mediated transcription and apoptosis by exportin-1 (XPO1) inhibition in mantle cell lymphoma. Cancer Sci.

[12] Andreeff M, Kelly KR, Yee K, Assouline S, Strair R, Popplewell L, Bowen D, Martinelli G, Drummond MW, Vyas P, Kirschbaum M, Iyer SP, Ruvolo V (2016). Results of the Phase I Trial of RG7112, a Small-Molecule MDM2 Antagonist in Leukemia. Clin Cancer Res.

[13] Chen C, Garzon R, Gutierrez M, Jacoby MA, Brown P, Flinn IW, Stone RM, Savoie ML, Baz R, Gabrail NY, Wang M, Martin P, Siegel DS (2015). Safety, efficacy, and determination of the recommended phase 2 dose for the oral selective inhibitor of nuclear export (SINE) selinexor (KPT-330). Blood.

[14] McCormack E, Haaland I, Venås G, Forthun RB, Huseby S, Gausdal G, Knappskog S, Micklem DR, Lorens JB, Bruserud O, Gjertsen BT (2012). Synergistic induction of p53 mediated apoptosis by valproic acid and nutlin-3 in acute myeloid leukemia. Leukemia.

[15] Kojima K, Kornblau SM, Ruvolo V, Dilip A, Duvvuri S, Davis RE, Zhang M, Wang Z, Coombes KR, Zhang N, Qiu YH, Burks JK, Kantarjian H (2013). Prognostic impact and targeting of CRM1 in acute myeloid leukemia. Blood.

[16] Aziz MH, Shen H, Maki CG (2011). Acquisition of p53 mutations in response to the non-genotoxic p53 activator Nutlin-3. Oncogene.

[17] Michaelis M, Rothweiler F, Barth S, Cinatl J, van Rikxoort M, Löschmann N, Voges Y, Breitling R, von Deimling A, Rödel F, Weber K, Fehse B, Mack E (2011). Adaptation of cancer cells from different entities to the MDM2 inhibitor nutlin-3 results in the emergence of p53-mutated multi-drug-resistant cancer cells. Cell Death Dis.

[18] Beà S, Valdés-Mas R, Navarro A, Salaverria I, Martín-Garcia D, Jares P, Giné E, Pinyol M, Royo C, Nadeu F, Conde L, Juan M, Clot G (2013). Landscape of somatic mutations and clonal evolution in mantle cell lymphoma. Proc Natl Acad Sci USA.

[19] Donehower LA (2014). Phosphatases reverse p53-mediated cell cycle checkpoints. Proc Natl Acad Sci USA.

[20] Lu X, Nguyen TA, Moon SH, Darlington Y, Sommer M, Donehower LA (2008). The type 2C phosphatase Wip1: an oncogenic regulator of tumor suppressor and DNA damage response pathways. Cancer Metastasis Rev.

[21] Yang H, Gao XY, Li P, Jiang TS (2015). PPM1D overexpression predicts poor prognosis in non-small cell lung cancer. Tumour Biol.

[22] Liu S, Qi L, Han W, Wan X, Jiang S, Li Y, Xie Y, Liu L, Zeng F, Liu Z, Zu X (2014). Overexpression of wip1 is associated with biologic behavior in human clear cell renal cell carcinoma. PLoS One.

[23] Lambros MB, Natrajan R, Geyer FC, Lopez-Garcia MA, Dedes KJ, Savage K, Lacroix-Triki M, Jones RL, Lord CJ, Linardopoulos S, Ashworth A, Reis-Filho JS (2010). PPM1D gene amplification and overexpression in breast cancer: a qRT-PCR and chromogenic *in situ* hybridization study. Mod Pathol.

[24] Hirasawa A, Saito-Ohara F, Inoue J, Aoki D, Susumu N, Yokoyama T, Nozawa S, Inazawa J, Imoto I (2003). Association of 17q21-q24 gain in ovarian clear cell adenocarcinomas with poor prognosis and identification of PPM1D and APPBP2 as likely amplification targets. Clin Cancer Res.

[25] Gilmartin AG, Faitg TH, Richter M, Groy A, Seefeld MA, Darcy MG, Peng X, Federowicz K, Yang J, Zhang SY, Minthorn E, Jaworski JP, Schaber M (2014). Allosteric Wip1 phosphatase inhibition through flap-subdomain interaction. Nat Chem Biol.

[26] Esfandiari A, Hawthorne TA, Nakjang S, Lunec J (2016). Chemical Inhibition of Wild-Type p53-Induced Phosphatase 1 (WIP1/PPM1D) by GSK2830371 Potentiates the Sensitivity to MDM2 Inhibitors in a p53-Dependent Manner. Mol Cancer Ther.

[27] Richter M, Dayaram T, Gilmartin AG, Ganji G, Pemmasani SK, Van Der Key H, Shohet JM, Donehower LA, Kumar R (2015). WIP1 phosphatase as a potential therapeutic target in neuroblastoma. PLoS One.

[28] Pechackova S, Burdova K, Benada J, Kleiblova P, Jenikova G, Macurek L (2016). Inhibition of WIP1 phosphatase sensitizes breast cancer cells to genotoxic stress and to MDM2 antagonist nutlin-3. Oncotarget.

[29] Basso K, Margolin AA, Stolovitzky G, Klein U, Dalla-Favera R, Califano A (2005). Reverse engineering of regulatory networks in human B cells. Nat Genet.

[30] Rosenwald A, Wright G, Wiestner A, Chan WC, Connors JM, Campo E, Gascoyne RD, Grogan TM, Muller-Hermelink HK, Smeland EB, Chiorazzi M, Giltnane JM, Hurt EM (2003). The proliferation gene expression signature is a quantitative integrator of oncogenic events that predicts survival in mantle cell lymphoma. Cancer Cell.

[31] Beckerman R, Prives C (2010). Transcriptional regulation by p53. Cold Spring Harb Perspect Biol.

[32] Drakos E, Atsaves V, Li J, Leventaki V, Andreeff M, Medeiros LJ, Rassidakis GZ (2009). Stabilization and activation of p53 downregulates mTOR signaling through AMPK in mantle cell lymphoma. Leukemia.

[33] Lioni M, Noma K, Snyder A, Klein-Szanto A, Diehl JA, Rustgi AK, Herlyn M, Smalley KS (2008). Bortezomib induces apoptosis in esophageal squamous cell carcinoma cells through activation of the p38 mitogen-activated protein kinase pathway. Mol Cancer Ther.

[34] Dasmahapatra G, Patel H, Friedberg J, Quayle SN, Jones SS, Grant S (2014). *In vitro* and *in vivo* interactions between the HDAC6 inhibitor ricolinostat (ACY1215) and the irreversible proteasome inhibitor carfilzomib in non-Hodgkin lymphoma cells. Mol Cancer Ther.

[35] Luster TA, Mukherjee I, Carrell JA, Cho YH, Gill J, Kelly L, Garcia A, Ward C, Oh L, Ullrich SJ, Migone TS, Humphreys R (2012). Fusion toxin BLyS-gelonin inhibits growth of malignant human B cell lines *in vitro* and *in vivo*. PLoS One.

[36] Bain J, Plater L, Elliott M, Shpiro N, Hastie CJ, McLauchlan H, Klevernic I, Arthur JS, Alessi DR, Cohen P (2007). The selectivity of protein kinase inhibitors: a further update. Biochem J.

[37] Ray-Coquard I, Blay JY, Italiano A, Le Cesne A, Penel N, Zhi J, Heil F, Rueger R, Graves B, Ding M, Geho D, Middleton SA, Vassilev LT (2012). Effect of the MDM2 antagonist RG7112 on the P53 pathway in patients with MDM2-amplified, well-differentiated or dedifferentiated liposarcoma: an exploratory proof-of-mechanism study. Lancet Oncol.

[38] Khoo KH, Verma CS, Lane DP (2014). Drugging the p53 pathway: understanding the route to clinical efficacy. Nat Rev Drug Discov.

[39] Yamaguchi H, Durell SR, Chatterjee DK, Anderson CW, Appella E (2007). The Wip1 phosphatase PPM1D dephosphorylates SQ/TQ motifs in checkpoint substrates phosphorylated by PI3K-like kinases. Biochemistry.

[40] Chou TC, Talalay P (1984). Quantitative analysis of dose-effect relationships: the combined effects of multiple drugs or enzyme inhibitors. Adv Enzyme Regul.

