# Supplementary material for: The pathophysiological significance of PPM1D and therapeutic targeting of PPM1D-mediated signaling by GSK2830371 in mantle cell lymphoma
Source: Oncotarget. 2016 Sep 8;7(43):69625–37. doi: 10.18632/oncotarget.11904 (PMC5342503; doi:10.18632/oncotarget.11904)
Supplement: Supplementary file 1 [file oncotarget-07-69625-s001.pdf]

The pathophysiological significance of PPM1D and therapeutic targeting of PPM1D-mediated signaling by GSK2830371 in mantle cell lymphoma

Supplementary Materials

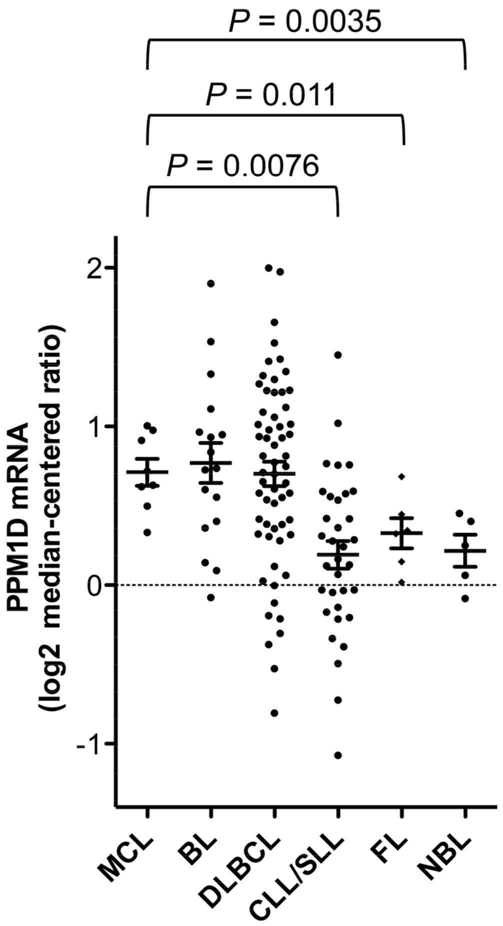

Supplementary Figure S1: PPM1D mRNA levels across major lymphoma types. Normal B-lymphocytes (NBL) are included for comparison. MCL, mantle cell lymphoma; BL, Burkitt's lymphoma; DLBCL, diffuse large B-cell lymphoma; CLL/SLL, chronic lymphocytic leukemia/small lymphocytic lymphoma; FL, follicular lymphoma.

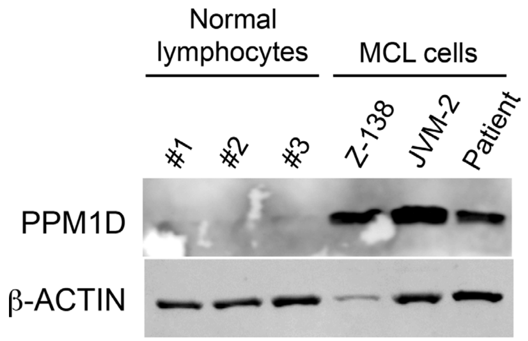

Supplementary Figure S2: PPM1D expression in normal lymphocytes, mantle cell lymphoma (MCL) cell lines and primary MCL cells from a patient with MCL in leukemic phase.

A

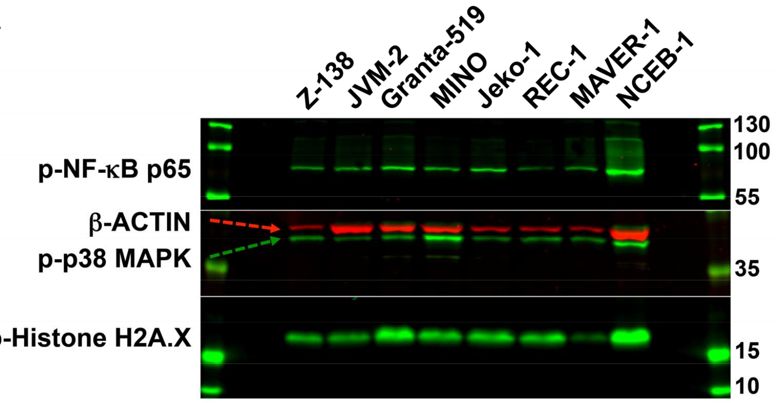

B

		Correlation coefficient (r)	Probability (P)
p-NF-kB	48 hours	-0.15	0.73
	72 hours	0.30	0.47
p-p38 MAPK	48 hours	0.10	0.81
	72 hours	0.12	0.77
p-Histone H2A.X	48 hours	–0.09	0.84
	72 hours	0.27	0.52

Supplementary Figure S3: GSK2830371-induced phosphorylated protein levels of PPM1D targets do not determine the sensitivity of mantle cell lymphoma (MCL) cells to GSK2830371. (**A**) GSK2830371-induced phosphorylated protein levels of PPM1D targets in MCL cell lines. (**B**) Correlation coefficient and probability values of the 48-hour and 72-hour growth inhibitory effects of 10 μM GSK2830371 relative to PPM1D target proteins.

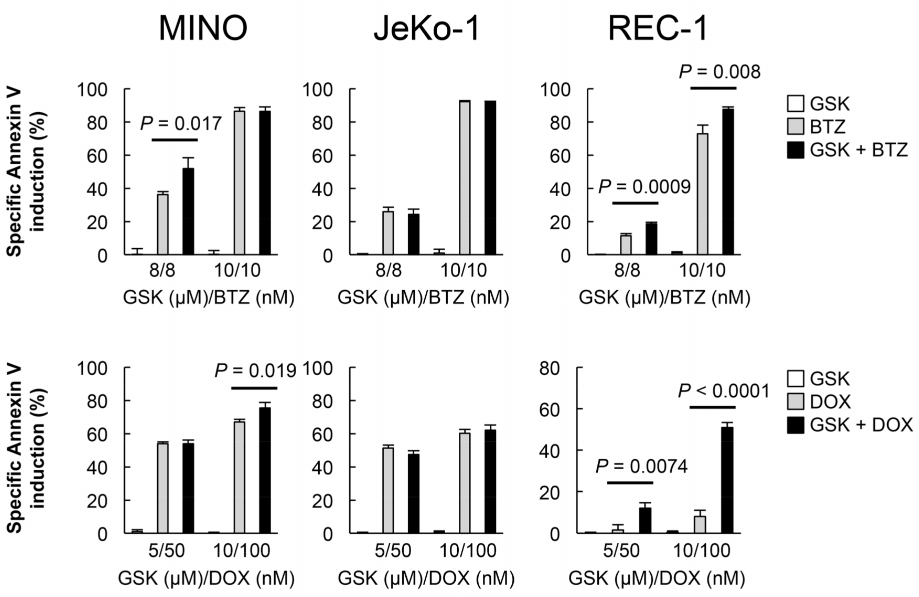

Supplementary Figure S4: Potentiation effect of GSK2830371 on bortezomib- and doxorubicin-induced apoptosis in p53-defective mantle cell lymphoma cells. Cells were treated for 72 hours with GSK2830371 (GSK) and bortezomib (BTZ), or doxorubicin (DOX) either as individual agents or in combination, after which the annexin V-positive fractions were determined.

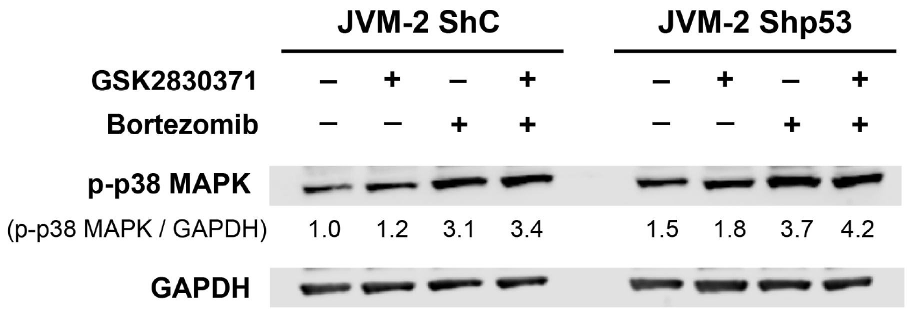

Supplementary Figure S5: GSK2830371 and Bortezomib synergistically increase levels of phosphorylated p38 MAPK, irrespective of p53 status. (A) JVM-2 cells transduced with lentivirus encoding either control shRNA (ShC) or p53-specific shRNA (Shp53) were treated for 24 hours with 10 μM GSK2830371 or 14 nM bortezomib either as individual agents or in combination, after which the levels of phosphorylated p38 MAPK were determined. The intensities of phosphorylated p38 MAPK signals were quantified, the intensities relative to GAPDH were calculated, and the levels in untreated ShC cells were set at 1.0. The results are representative of three independent experiments.
